# CT and MRI manifestations of mediastinal cavernous hemangioma and a review of the literature

**DOI:** 10.1186/s12957-019-1742-1

**Published:** 2019-12-04

**Authors:** Yu Bai, Guoshu Zhao, Yongming Tan

**Affiliations:** 1 Speciality of Medical Imaging, Nanchang University, Nanchang, 330006 People’s Republic of China; 2 Department of Radiology, The First Affiliated Hospital of Nanchang University, Nanchang, 330006 People’s Republic of China

**Keywords:** Mediastinal cavernous hemangioma, Tomograph, MRI

## Abstract

**Background:**

The cavernous hemangioma of mediastinum (CHM) is a rare benign lesion caused by congenital vascular dysplasia. However, its incidence is extremely low, and patients often lack relevant clinical symptoms. So we analyzed retrospectively some cases to investigate the imaging features of cavernous hemangioma of mediastinum (CHM) and improve the diagnostic accuracy.

**Methods:**

The CT/MRI imaging features and clinical information of 19 patients with CHM were analyzed retrospectively.

**Results:**

The lesions of 18 CHM patients were single. Twelve cases in the anterior mediastinum and 8 in the posterior mediastinum. The diameter of CHM ranges from 2.0 to 7.0 cm. Thirteen cases were oval-shaped or round, 4 cases were lobulated, and 2 cases were irregular. Phleboliths or nodular calcification were identified in four cases. High signal of T2WI lipid suppression in two cases and blood vessel shadows were observed in two cases. After contrast-enhanced scan, the nodular enhancement of arteries were identified in 14 cases and contrast agent was further filled of the venous phase, where “fast in and slow out” feature was performed. One case showed inhomogeneous enhancement, one case performed “fast in and slow out” feature of multiphase-enhanced MRI. Besides, aberrant veins can be seen in or around the lesion among five cases.

**Conclusions:**

CHM is more frequently located at the anterior mediastinum than at the posterior mediastinum. The performance of phleboliths, high signal on T2WI fat suppression and DWI, the nodular enhancement of the artery, venous and delayed phase filling, enhanced “fast in and slow out,” and aberrant veins in the lesion are helpful for the diagnosis and differential diagnosis. Multiple period contrast-enhanced CT and MRI scan is helpful for the diagnosis of CHM.

## Introduction

The cavernous hemangioma of mediastinum (CHM) is a rare disease of mediastinal benign lesions that originates from isolated embryonic simple angioblast tissue or vascular dysplasia, which is composed of masses with varying sizes and dilated cavernous sinus pathologically. Its incidence rate is below 0.5% in mediastinal lesions from some literatures [1] and patients often lack of relevant clinical symptoms [1]. Therefore, the image manifestation is diverse and the rate of misdiagnosis before surgery is high. The author analyzed the clinical performance and imaging findings of 19 cases that surgically confirmed CHM and combined with literature reviews to improve the understanding of CHM and diagnostic accuracy.

## Materials and methods

### Clinical presentation

Nineteen patients with CHM confirmed by pathology from January 2011 to March 2018 were collected. There were 6 males and 13 females with a male to female ratio of 1:2.1 and an age of 21 to 75 years. Nine cases showed clinical symptoms such as chest tightness, back pain, and cough and one case with binocular fatigue, whereas nine cases were found accidently during health physical examination. There were no endocrine abnormalities in all cases.

### Diagnostic tool

Fifteen patients underwent CT plain scan and enhancement, two patients underwent CT enhancement directly, one patient underwent both CT and MRI plain scan and enhancement, and one patient underwent CT plain scan and MRI plain scan plus enhancement. The CT scan used was the Somatom Definition AS+ model. In enhanced scanning, non-ionic contrast agent iohexol (300 mg/ml) 100~125 ml was used, with a flow rate of 3.0~3.5 ml/s through the ulnar vein bolus injection, 25~30 s after the injection of contrast agent, and finally acquired arterial and venous phase. MRI scan were performed using a Siemens 3.0 T Magnetom Trio Tim magnetic resonance system and 16-channel skull coil. Enhanced scanning through ulnar vein injection of Magnevist solution at a dose of 0.3 mmol/kg, with a rate of 2.0 to 3.0 ml/s. Arterial phase (20~30 s), venous phase (60 s), delayed phase (120~180 s), and after-delayed phase (200 s) scanning were also acquired.

### Image analysis

The imaging data of all patients were diagnosed blindly by two senior radiologists to analyze the location, size, shape, boundary, density, and signal and enhanced the mode value of the lesions. After enhancement, the average CT value of lesions was slightly enhanced from 0 to 20 Hu, moderately enhanced from 20 to 40 Hu, and significantly enhanced which exceeded 40 Hu.

### Pathological examination

The obtained specimens were fixed with 10% formaldehyde solution, routine embedment, paraffin sections, hematoxylin-eosin staining (HE staining), and MaxVision/HRP immunohistochemical staining. The detection process of immunohistochemistry is as follows: first, after dewaxing paraffin section to water, 3% H_2_O_2_ inhibited endogenous peroxidase for 15 min and PB was washed three times for 3 min. Secondly, according to the requirement of primary antibody, the tissue antigen was correspondingly repaired (citric acid buffer 95–100 for 10 min or trypsinase digestion for 10 min); PB was washed three times for 3 min. Thirdly, specific primary antibody (negative control replacing primary antibody with PBS) is washed at room temperature for 1 hour or 40 °C overnight; PB is washed three times for 3 min. HRP-labeled streptavidin is washed at room temperature for 15 min; PB is washed three times for 3 min. And then DAB color developed after 3 min, finally after flow washing, hematoxylin re-staining, being dehydrated, and applying transparent and neutral gum seals.

## Results

### The quantities, locations, sizes, shapes, and boundaries of lesions

Eighteen cases were solitary lesions and one was multiple. The sizes of masses were about 2.0 to 7.0 cm. There were 12 cases of anterior mediastinum and 7 cases of posterior mediastinum. Thirteen cases were round or oval-shaped, 4 cases were lobulated, and 2 cases were irregular. The boundaries were clear in 15 cases, and the remaining 4 cases were unclear with the mediastinal pleura and the intraspinal spinal meninges.

### CT performance

**Fig. 1:**
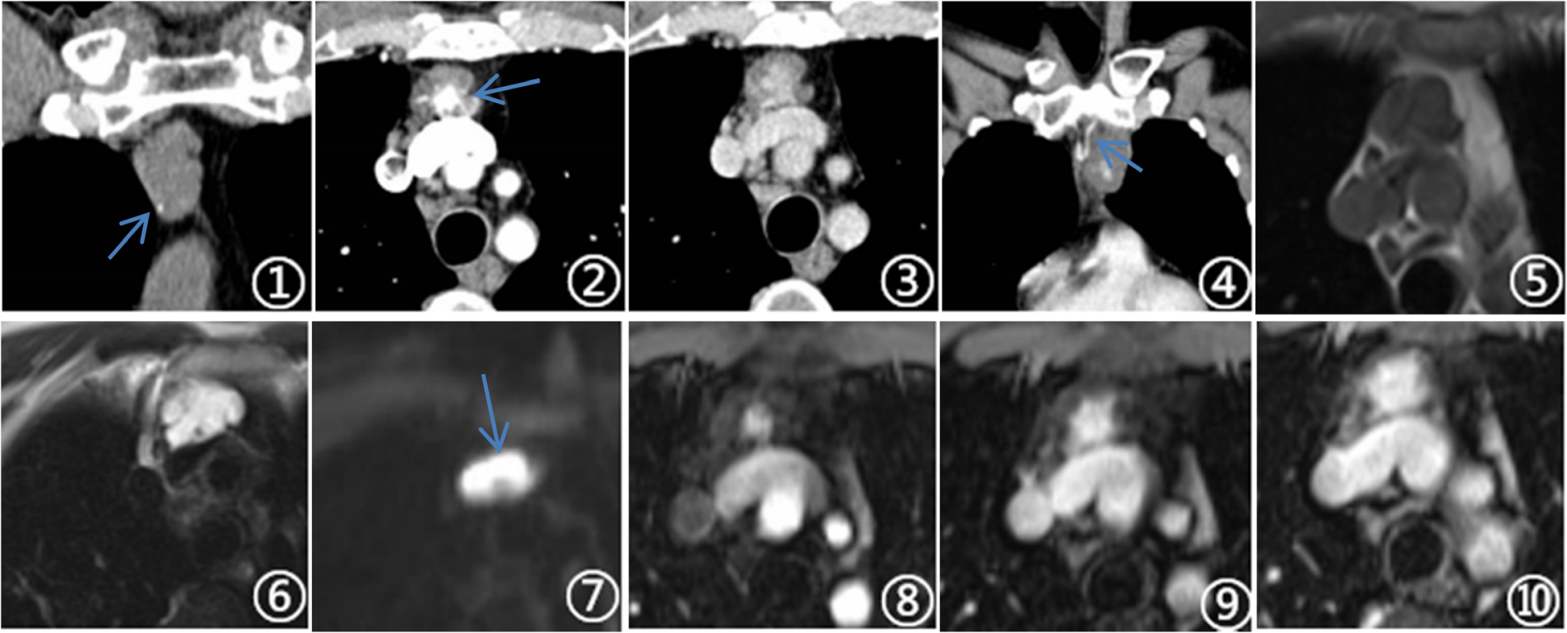
**1-10** were the same case. **1** Lesion has equal density and small circular calcifications were observed inside. **6-7** Mass on the fat suppression and DWI (*b* = 800) showed obviously high signal with small blood vessel shadows inside. **2-3** and **7-10** In the arterial phase, in the center of the lesions, there is the nodular enhancement. As time goes by, the enhanced range was expanded and showed a “fast in and slow out” performance. **4, 8** In the arterial phase, aberrant draining veins filled by the contrast agent antidromic could be observed, which were connected to the left brachiocephalic vein

**Fig. 2:**
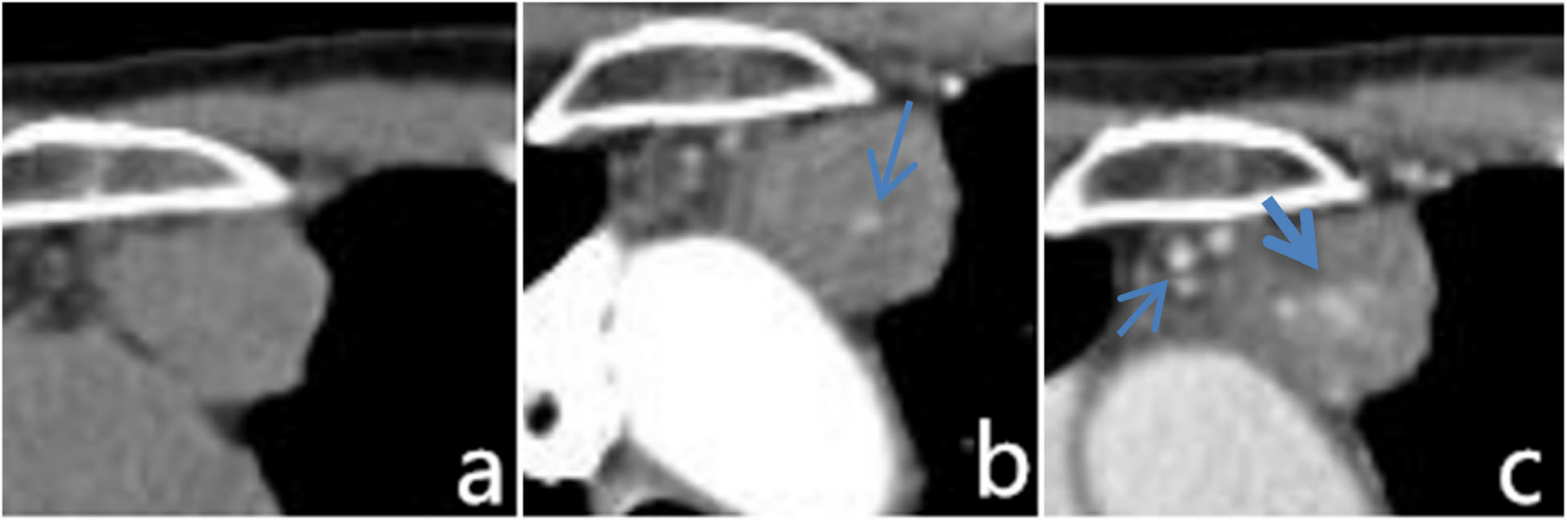
**a-c** Visible soft tissue mass with uniform density in the anterior superior mediastinum. In enhanced scanning, nodular enhancement can be seen in the center of the arterial phase. The range of enhancement in the venous phase was expanded which showed a “fast in and slow out” performance

CT plain scan in 16 cases showed uniform equi-density (compared with the same layer of muscle), with CT value of 20~46 Hu (average 31 Hu). Small circular phleboliths or nodular calcification were visible in four lesions (Fig. 1 (1)), and no obvious calcification or phleboliths in the remaining 15 lesions. When enhanced scanning was conducted, 14 cases were mildly enhanced, three cases were moderately enhanced, and two cases were significantly enhanced. Enhanced nodules in the center or periphery of lesions were visible in the arterial phase of 14 cases, and the degree of enhancement was similar to the same layer of thoracic aorta. In the venous phase, lesions continued to enhance further and the enhanced range was expanded (Fig. 1 (1, 2, and 3) and Figure 2a–c). Five cases in the anterior mediastinum revealed aberrant draining veins that antidromic filled by contrast agent after enhanced scanning (Fig. 1 (4)).

### MRI performance

Two cases of MRI scan showed equal T1WI signal, slightly longer T2WI signal with inhomogeneous signal inside, obviously high signal of T2WI fat suppression (Fig. 1 (6)), and high signal of DWI (*b* = 800) (Fig. 1 (7)). The blood vessel shadows were observed in one lesion (Fig. 1 (6)). One lesion was unclear with the adjacent mediastinal pleura. One case of enhanced scanning showed nodular enhancement in the arterial phase, the contrast agent was further filled, and the enhanced range was expanded in the venous and delayed phases, which performed typical “fast in and slow out” feature. And aberrant drainage veins connecting to the left brachiocephalic vein were observed (Fig. 1 (8–10)). One case in the arterial phase emerged mild and inhomogeneous annular enhanced, with continuous enhancement in the venous and delayed phases, and enhancement intensity was increased.

### Surgery, pathology, and immunohistochemistry

**Fig. 3:**
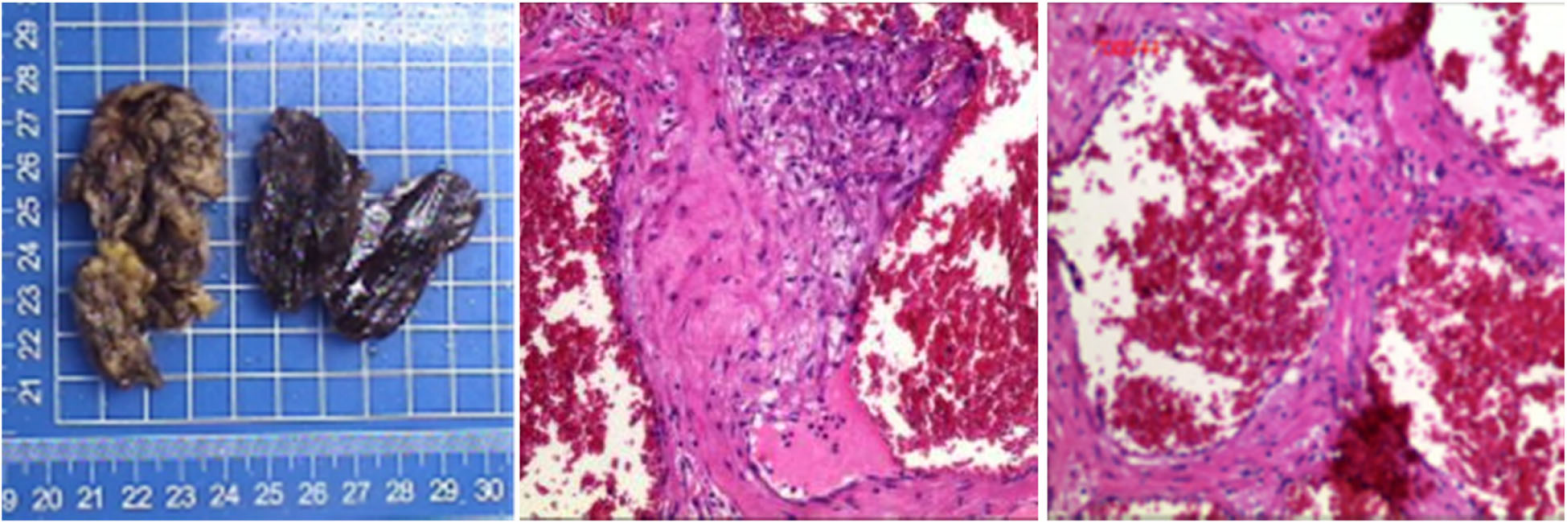
The pathological lesion was generally grayish red and grayish yellow and the surface was similar to an envelope. The cut surface was grayish and spongy. The blood vessels were rich under the microscope. The lumen size was different, the wall thickness was uneven, and the vascular smooth muscle hyperplasia was obvious. Part of the sponge-like part of the vine has congestion in the lumen diagnosed as (pre-upper mediastinum) cavernous hemangioma (HE × 40)

Nineteen lesions underwent surgical resection and the pathological specimens were confirmed to be cavernous hemangioma. The lesion was composed of masses with varying sizes and dilated cavernous sinus. Its cut surface was grayish brown and spongy with rough edges. A large number of irregular blood sinus and red blood cells were seen under the microscope with peripheral tissue fibrosis and smooth muscle hyperplasia. There were many different sizes of cystic vessels and red blood cells in the proliferated fibrous tissues and the cystic vessels presented sponge-like changes. Meanwhile, the blood sinus cavity was congestive or filled with some calcified thrombus. Immunohistochemistry showed SMA (+), S100 (−), DES (−), MBP (−), and CD34 (vascular +). (SMA is a smooth muscle marker, and CD34 is a mesenchymal origin that is used in the diagnosis of vascular lesions to indicate the origin of vascular lesions (Fig 3)).

## Discussion

CHM is a rare disease of mediastinal benign lesion originating from vascular endothelial cells which is caused by abnormal vascular development during embryonic period and composed of masses with varying sizes and dilated cavernous sinus pathologically. The inner wall is lined with vascular endothelium, and the blood sinus cavity is congestive or filled with some phleboliths (calcified thrombosis) [1]. Eighteen cases in this group (18/19) were solitary lesions, and few multiple cases were reported in the previous studies, which may be the characteristic of this disease. The literature reports that the average age of CHM is about 35 years old [2, 3]. Most of the patients in this group were middle- and old-aged patients over 50 years old, which may be related to the non-specific clinical symptoms and delayed treatment. Reports showed that there was no significant gender difference in CHM patients, and the proportion of female patients in this group was about 68.4% (13/19), which was different from previous reports that may be related to the statistical bias of small sample size [1, 2, 4]. Most patients with CHM lack specific clinical manifestations [2], mainly related to the locations, sizes, and invasion with adjacent structures of lesions [1, 4]. In this group, the clinical symptoms of nine patients were chest tightness, chest pain, back pain, and cough, and one case manifested fatigue in both eyes, and they all could be considered as relatively clinical symptoms caused by the lesions which compressed and stimulated adjacent structures. The remaining nine patients had no clinical symptoms of the chest and were diagnosed accidentally during health physical examination.

CHM could occur in any area of the mediastinum. In general, the lesion can occur in any part of the mediastinum, with most of the anterior superior mediastinum (about 70%) and the second in the posterior mediastinum (20%) [5]. In this group, 12 cases occurred in the anterior mediastinum (12/19), while the remaining 7 cases occurred in the posterior mediastinum, which was similar to previous reports. Most of the CHM patients show clear borders and were oval-shaped or round, but it can also invade neighboring tissues and organs to be unclear with surrounding tissues. In this group, four lesions were unclear with adjacent pleura which may be related to the lesions originating from the intercostal blood vessels, and these lesions were easy to misdiagnose as suspicious malignant lesions before operation in this condition. There was no report about this in the past. Reports showed that the intralesional phlebolith is a characteristic imaging manifestation of CHM, and CT was more likely to find it, but the incidence is low. In previous studies, about 16–18% [2] phleboliths were visible in CHM. In this group, four cases (4/19) of CT scanning showed small circular phleboliths or nodular calcifications, which was similar to previous reports. In this group, two cases with MRI showed equal signal on T1WI, slightly longer signal on T2WI, and obviously high signal on T2WI fat suppression. Some scholars believe that high signal on T2WI fat suppression may be an important sign of CHM [6]. Besides, two lesions in this group showed high signal on DWI (*b* = 800), which may be a characteristic image of CHM and be of great significance for the diagnosis of CHM. This sign has not been reported in the past and may be related to full sinusoids in pathology, scattered hemorrhage, and diffusing limitation of CHM. Due to the vascular sinus interstitial tissue in CHM, there was obviously hyperplastic and fibrotic smooth muscle tissue and there may be different degrees of thrombosis in the vascular sinus cavity so that the blood flow pattern is variable, which leads to more complicated and changeable enhanced ways [4, 7]. Most of the CHM (14/19) in this group showed mild uneven enhancements while three cases were moderately enhanced and two cases were obviously unevenly enhanced, which was basically consistent with previous reports [2]. At the same time, in 14 cases of CHM, clearly intensified nodules were observed in the peripheral or center of the lesions during CT-enhanced scanning. The degree of enhancement was similar to the same layer of the thoracic aorta. As continuously enhanced in the venous phase, the enhancement range was expanded characteristically, which was similar to the progressive feature of hepatic cavernous hemangioma. And the MRI multiphase-enhanced scan of one lesion appeared in the “fast in and slow out” feature more clearly, which can be regarded as a specifically diagnostic basis for CHM and be of great value for the diagnosis and identification of this disease. The enhanced feature has been reported in the past [8]; however, it is not common to show this sign, which may be related to the time of the enhanced scanning for different researches. In this group, the ulnar vein bolus and dual-phase scanning project (25 s arterial phase and 60 s venous phase after contrast agent injection) was used during the chest CT-enhanced scanning and multiphase was used during MRI-enhanced scanning (25 s, 60 s, 150 s, and 200 s, after contrast agent injection). Previous studies generally used 30-s first phase and 120-s delayed phase scanning project after contrast agent was injected [9]. In addition, dilated malformed drainage veins connected with the brachiocephalic vein and superior vena cava in the lesions were found after contrast agent was injected in five cases of the anterior mediastinum, which was consistent with the feature found by some scholars [9, 10]. In this group, blood vessel shadows were found in T1WI of one lesion, yet this phenomenon has not been reported in the past. The discovery of these signs often indicates that the lesions is vascular, providing characteristic imaging features for its diagnosis and differential diagnosis, which can prevent patients from further invasive examination and play an important role in the development of surgical project.

## Conclusion

CHM is a benign disease in a rare site of common clinical diseases while the clinical manifestations are non-specific and easily misdiagnosed. In summary, the author considers that the following signs can contribute to the diagnosis of CHM:

- When the CT reveals a clear round or oval single-edged mass in the anterior-superior mediastinum that the enhanced scanning is mild to moderately unevenly enhanced, in this condition, we should put the possibility of CHM into consideration.
- If the lesions appear high signal on T2WI, obviously high signal on T2WI fat suppression, and high signal on DWI, it can further suggest the possibility of the disease.
- If there are characteristic phleboliths, blood vessel shadows, and malformed drainage veins in the mass, the diagnosis of CHM should be highly indicated.
- If the feature is observed as follows: in the obvious nodular enhancement of the internal or peripheral of the mass in the arterial phase, the expanded enhancement range of delayed scanning, and the presence of characteristic “fast in and slow out” feature, the diagnosis can be basically confirmed. For CT suspected CHM cases, CT dynamic enhanced scan and MRI are helpful for diagnosis.

## Data Availability

The dataset used and analyzed during the current study is available from the corresponding author on reasonable request.
